# Functional divergence of the synapsin family in glioblastoma: Synapsin III as a candidate differentiation-restoring node

**DOI:** 10.3389/fneur.2026.1976698

**Published:** 2026-10-12

**Authors:** Xiangyu Dai, Yujie Ji, Zheng Cai, Haixin Zeng, Yi Li, Haining Zhou, Xu Zhang, Bing Li

**Affiliations:** 1 Department of Neurosurgery, Jiangnan University Medical Center, Wuxi No.2 People's Hospital, Jiangnan University, Wuxi, China; 2 Clinical Medical Research Center, Jiangnan University Medical Center, Wuxi No.2 People's Hospital, Jiangnan University, Wuxi, China

**Keywords:** differentiation therapy, glioblastoma, glioblastoma stem cells, neuron-glioma synapse, Notch signaling, Syn3, synapsin, Synapsin III

## Abstract

Glioblastoma (GBM) remains an aggressive adult primary brain tumor despite surgery, radiotherapy and temozolomide-based chemotherapy, partly because glioblastoma stem cells (GSCs) retain self-renewal capacity, lineage plasticity and therapeutic resistance. Cancer neuroscience has shown that glioma cells receive activity-dependent neuronal signals and exploit neural circuits to support tumor growth and invasion. Synaptic proteins may therefore regulate both neuron-glioma communication and tumor-cell state. Synapsin I, II, and III share conserved N-terminal domains but differ in C-terminal architecture, developmental expression and physiological function. An emerging model of functional divergence in GBM is supported by a recent preclinical study comparing Synapsin III with Synapsin I and II. In the tested GSC models, increasing Synapsin III promoted a neuronal-like differentiated phenotype through a neuregulin 3 (NRG3)-JAG1-NOTCH1 mechanism, with reduced stemness and proliferative growth. This review integrates structural, physiological and GBM-focused evidence to examine how the developmental functions of Synapsin III may be repurposed in malignant stem-like cells. Independent replication, durable loss of tumorigenicity and tumor-selective delivery are priorities for translation. Synapsin III is a candidate differentiation-restoring node whose clinical relevance remains to be established.

## Introduction

Glioblastoma (GBM) is clinically characterized by diffuse infiltration, rapid recurrence and limited durability of current standard therapy. The Stupp regimen established radiotherapy plus concomitant and adjuvant temozolomide as a clinical backbone, but long-term survival remains uncommon (1). A major biological explanation is the persistence of glioblastoma stem cells (GSCs), which combine self-renewal, adaptive plasticity and resistance to cytotoxic stress (2). These features position GBM as a disease driven not only by malignant genetic alterations but also by dysregulated developmental cell states.

Cancer neuroscience has added a further layer to this model. Two independent studies in 2019 showed that glioma cells can receive glutamatergic synaptic input and electrically integrate into neural circuits, providing direct evidence that neuronal activity can provide growth-promoting signals for tumor cells (3, 4). Earlier work had shown that neuronal activity promotes high-grade glioma growth through activity-regulated secretion of neuroligin-3 (NLGN3) (5). Subsequent therapeutic experiments indicated that targeting this dependency can suppress high-grade glioma growth in preclinical models (5, 6). Together, these studies changed the question from whether neurons influence glioma to which neuronal and synaptic programs are functionally required in specific glioma states.

Synaptic proteins sit at the center of this shift. They may be expressed as markers of neuronal differentiation, co-opted as drivers of malignant synaptic communication, or reactivated as regulators of tumor-cell fate (7, 8). The synapsins are especially relevant because they are presynaptic phosphoproteins that regulate synaptic vesicle organization and neurotransmitter release (8, 9). Yet the three mammalian family members are not interchangeable. Synapsin III has a distinct C-terminal J domain, a different developmental expression profile and functions that extend beyond mature synaptic vesicle cycling (9, 10). These differences provide a plausible structural basis for divergent roles in glioma.

This review examines synapsin-family functions in adult GBM, using Synapsin III to connect developmental cell-state regulation with tumor biology. Evidence from other gliomas provides circuit-level context, but does not establish the same differentiation mechanism in adult non-GBM gliomas or pediatric low- and high-grade gliomas.

## Structural and physiological logic of synapsin-family divergence

The synapsin family comprises Synapsin I, Synapsin II and Synapsin III, encoded by the human genes *SYN1, SYN2* and *SYN3*, respectively. The five major isoforms conventionally illustrated are Ia, Ib, IIa, IIb and IIIa. All share N-terminal domains A, B and C, whereas the C-terminal domain combinations differ (Figure 1A) (8). Domain A contributes phosphorylation-sensitive lipid interactions; B is a linker; and C is the most conserved region and contributes to vesicle, actin and oligomerization interactions. In the conventional domain scheme, Ia contains A-B-C-D-E, Ib A-B-C-D-F, IIa A-B-C-G-H-E, IIb A-B-C-G-I, and IIIa A-B-C-J-E.

**Figure 1:**
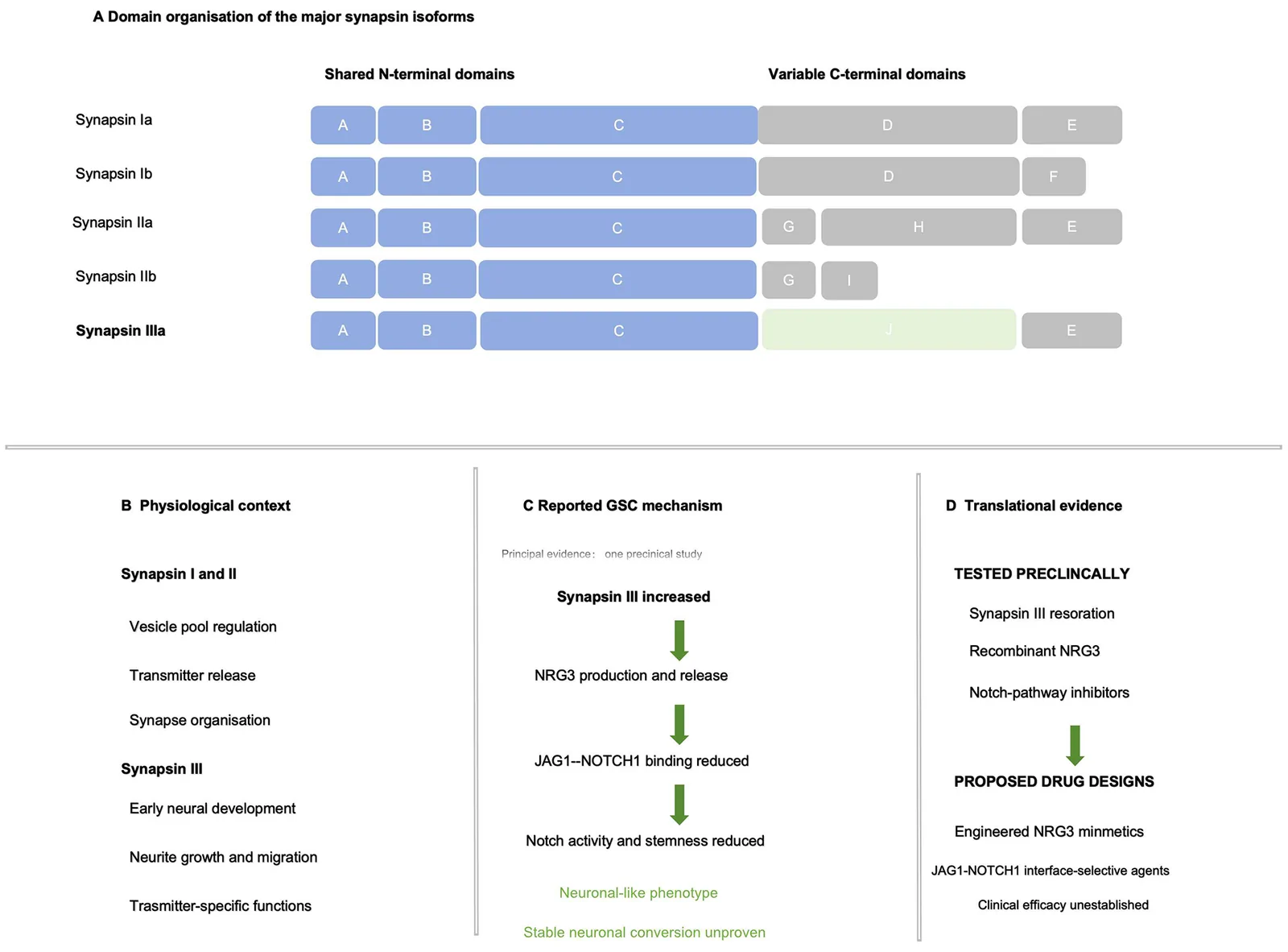
Synapsin-family organization and the emerging Synapsin III differentiation model in GBM. **(A)** Conventional domain organization of five major synapsin isoforms, drawn from N to C terminus. **(B)** Established neuronal functions provide physiological context. **(C)** Mechanistic model reported in the principal preclinical GSC study. **(D)** Evidence levels for translation.

Domain-level experiments in the squid giant presynaptic terminal support roles for domain A in late transmitter-release steps and for domains C and E in vesicle organization (8, 11). Synapsin IIIa contains the distinctive J domain, absent from the major Synapsin I and II isoforms, whereas domain E is shared by the three a-type isoforms (8, 9). The J domain offers a structural hypothesis for functional specificity, but its necessity for neurogenic or glioma-suppressive effects has not been demonstrated. Synapsin III is enriched in neurogenic regions and is detected beyond conventional presynaptic terminals, including neuronal cell bodies and growth-associated compartments (10). These localization patterns argue against treating it simply as a late presynaptic-vesicle marker.

Physiological studies further support non-redundancy among synapsin isoforms. Synapsin III has been implicated in neurotransmitter release, but its most relevant functions for glioma biology may lie in early neuronal development and cell migration rather than mature synaptic maintenance (12, 13). In adult hippocampal neurogenesis, Synapsin III is involved early in neural progenitor development (9, 13). It also acts downstream of semaphorin 3A/cyclin-dependent kinase 5 (CDK5) signaling to regulate radial migration and orientation of pyramidal neurons (14). These observations connect Synapsin III to cell-fate and migration programs that are closer to GSC biology than to stable mature synapses.

The physiological contrast with Synapsin I and II is important. Synapsin I and II are established regulators of presynaptic vesicle pools, synapse formation and synaptic plasticity (8, 15). Synapsin II can influence neurotransmitter release and presynaptic terminal formation (16, 17). Synapsin I phosphorylation is also linked to activity-dependent control of synapse formation (8, 18). Thus, physiological studies already support substantial functional specialization among synapsin family members. Figure 1B summarizes these physiological roles alongside the domain comparison in Figure 1A.

## Synapsin I and Synapsin II in glioma: suggestive but underdeveloped evidence

The functions of Synapsin I in GBM are less well characterized than its neuronal functions. Deng and colleagues compared overexpression of the three synapsin family members in patient-derived GSC cultures (19). In the tested models, increasing Synapsin I did not reproduce the reductions in proliferation, Nestin protein abundance or sphere formation observed with Synapsin III. Its effects in other GBM states remain unresolved.

Further gain- and loss-of-function studies should test whether manipulating *SYN1* affects GSC self-renewal, neuronal-like differentiation, synaptic input or treatment response. Its neuronal-lineage association motivates evaluation as a differentiation marker, although its biomarker value in GBM remains unvalidated.

Synapsin II also warrants investigation because of its established roles in neurotransmitter release and presynaptic terminal formation (16, 17). The same GSC study found that Synapsin II overexpression did not reproduce the tested suppressive effects of Synapsin III (19). This initial negative comparison does not resolve context-dependent activity, isoform-specific functions or the consequences of endogenous *SYN2* depletion.

Current evidence therefore supports an asymmetry in the depth of mechanistic characterization. Initial overexpression comparisons are available for all three family members, but the NRG3-JAG1-NOTCH1 mechanism and differentiation-associated suppression of GSCs have been developed principally for Synapsin III within one study (19). Independent replication and matched loss-of-function and rescue experiments are still needed before this pattern can be generalized.

Taken together, the available evidence supports an emerging model of synapsin-family divergence in glioma. The physiological differences among the proteins are well established, but they do not by themselves predict tumor function. Synapsin III currently provides the clearest experimental link to GSC state control, whereas the roles of Synapsin I and II remain incompletely characterized.

## Synapsin III in GBM: from low expression to differentiation-restoring function

The strongest mechanistic evidence for a synapsin-family member in GBM comes from Deng and colleagues' 2025 study (19). Analyses of glioma transcriptomic datasets associated higher *SYN3* mRNA abundance with better survival and reported lower transcript levels in GBM than in other glioma contexts. These transcript-level associations may reflect cellular composition and differentiation state in bulk tissue; they do not directly measure Synapsin III protein abundance.

In patient-derived GSCs, forced *SYN3* expression increased Synapsin III protein abundance. Immunoblotting and immunostaining showed increased neuronal markers, including microtubule-associated protein 2 (MAP2) and neuronal nuclei antigen (NeuN), and reduced Nestin, accompanied by lower proliferation and sphere-forming capacity (19). Together, these changes support a neuronal-like differentiated phenotype. Marker changes, neurite-like morphology and reduced sphere formation do not by themselves demonstrate complete, stable neuronal transdifferentiation or exclude contributions from growth arrest. Establishing durable functional conversion would require lineage-resolved follow-up after withdrawal of the inducing signal, neuronal electrophysiology and persistent loss of tumor-initiating capacity.

The reported mechanism links Synapsin III to extracellular control of Notch signaling (Figure 1C). Increasing Synapsin III enhanced neuregulin 3 (NRG3) expression and release. NRG3 bound JAG1, the membrane-associated ligand Jagged1, and interfered with its engagement of the NOTCH1 receptor (19). In canonical signaling, ligand engagement enables receptor cleavage and release of the Notch intracellular domain (NICD), which regulates transcription with nuclear partners, including RBPJ and MAML (20). Reduced NICD and HES1 protein abundance in the GSC study therefore provides pathway-level evidence downstream of the NRG3-JAG1 interaction (19). HES1 is a Notch-responsive transcriptional effector; Nestin, MAP2 and NeuN instead report stem-like or neuronal phenotypes, while proliferation and sphere formation assess related functional outcomes. The molecular steps coupling Synapsin III to NRG3 production and release, and the endogenous signals that regulate this coupling in GBM, remain unresolved.

Other differentiation-directed approaches in GBM offer complementary routes to altering tumor-cell state. Forced expression of the proneural transcription factor ASCL1 can promote neuronal fate and suppress GSC tumorigenicity through chromatin reorganization (21). Bone morphogenetic protein 4 (BMP4) reduced the tumor-initiating potential of human GBM-derived cells in preclinical models (22). Recombinant human BMP4 subsequently reached a first-in-human phase 1 study using convection-enhanced delivery in 15 patients with recurrent GBM. The study supported feasibility and tolerability and reported tumor responses in a subset, but its small, uncontrolled design does not establish comparative efficacy (23). BMP4 thus provides a clinically explored differentiation-oriented comparator, whereas ASCL1-mediated reprogramming and Synapsin III restoration remain preclinical approaches. Synapsin III is distinguished by the reported extracellular NRG3-JAG1 interaction (19).

Acute promyelocytic leukemia (APL) provides a useful, but mechanistically distinct, precedent (24, 25). Its characteristic PML-RARA fusion oncoprotein imposes a myeloid differentiation block. All-trans retinoic acid (ATRA) acts through the RARA portion, whereas arsenic trioxide targets the PML portion; their complementary actions promote oncoprotein degradation as well as relief of differentiation arrest. Experimental APL work showed that differentiation can be uncoupled from elimination of leukemia-initiating cells, highlighting loss of self-renewal as a separate requirement for durable disease control (25). A randomized trial established the efficacy of ATRA plus arsenic trioxide in low-to-intermediate-risk APL (26). PML-RARA is a disease-driving target, whereas Synapsin III is a proposed regulator of tumor-cell state. GBM lacks an established shared dependency on this differentiation-restoring axis and contains heterogeneous, plastic cell populations (2). Infiltration, drug delivery across brain barriers and reversion to stem-like states further complicate translation. For GBM, durable suppression of tumor initiation is therefore a critical endpoint alongside differentiation markers.

## How Synapsin III fits into cancer neuroscience

Synapsin III biology intersects with the neuron-glioma synapse model, in which neuronal input drives depolarization, calcium signaling and proliferation (3, 4). Glioma synapses also recruit adaptive plasticity mechanisms (27). These growth-promoting interactions provide the context for examining differentiation-associated effects of individual synaptic proteins.

Findings on Synapsin III link a neuronal-like phenotype to reduced stemness (19). Its reported effects therefore warrant distinction from the growth-promoting consequences of malignant circuit integration.

Neuron-glioma communication is broader than glutamatergic excitation. Gamma-aminobutyric acid (GABA)-mediated neuron-to-glioma synapses have been reported in diffuse midline glioma (28). Long-range cholinergic input has also been shown to promote GBM progression (29). In parallel, NLGN3-dependent signaling remains a major activity-regulated axis, and additional work has linked NLGN3 to cancer stem-cell maintenance and glioma progression (5, 6, 30). These studies show that malignant gliomas can sample multiple neurotransmitter and synaptic systems.

The synapsin family should be interpreted against this more complex circuit background. Synapsin III has developmental and neurotransmitter-specific functions (9, 12), making context-dependent tumor activity plausible. A key question is whether tumor cells with high *SYN3* mRNA abundance are simply associated with a more differentiated state, or whether increasing Synapsin III protein can impose a durable neuronal-like phenotype across clinically relevant GBM states.

## Network invasion, synaptic genes, and glioma-cell state

Circuit mapping places GBM within broader neuron-tumor networks (31). Human studies associate GBM-induced circuit remodeling with worse survival (32), indicating that neuronal connectivity is a clinically relevant disease feature.

GBM also exploits neuronal programs for invasion (33). Tumor microtubes coordinate intercellular communication and contribute to resistance in diffuse glioma (34). A Synapsin III-directed intervention would therefore need to address spatially organized invasion as well as cell-autonomous stemness.

Within infiltrated brain tissue, GBM cells increase Notch and synaptic-gene expression (35). Promoter-enhancer rewiring further links tumor regulatory states to neurogliomal communication (36). Synapsin activity should therefore be studied within these broader gene-regulatory and microenvironmental contexts.

The reported Synapsin III mechanism links a synaptic protein to the JAG1-NOTCH1 stemness pathway (19, 20). Its developmental roles (13, 14) motivate this connection, but do not establish that developmental programs are reinstated completely in malignant cells.

## Therapeutic implications and design constraints

Therapeutic targeting of malignant neural circuitry is being investigated. NLGN3 blockade and AMPA-type glutamate receptor (AMPAR)-related strategies have been proposed or tested in preclinical settings (6, 37). Perampanel, an AMPAR antagonist used as an antiseizure medication, is the subject of perioperative evaluation in progressive GBM in the PerSurge trial protocol (38). A separate pilot trial evaluated its effects on peritumoural hyperexcitability in newly diagnosed high-grade glioma (39). These studies evaluate circuit modulation and seizure-related outcomes.

Preclinical studies provide a basis for developing Synapsin III-directed interventions (Figure 1D). Deng and colleagues tested forced *SYN3* expression, recombinant NRG3 and pharmacological Notch-pathway inhibition in GSC models; intracranial adeno-associated virus serotype 9 (AAV9)-mediated *SYN3* delivery was also evaluated in tumor-bearing mice (19). These findings constitute preclinical proof of concept. By contrast, engineered NRG3 mimetics, JAG1-interface peptides and selective small molecules that reproduce the NRG3-JAG1 effect remain proposed approaches. Broad Notch-pathway inhibition is not equivalent to a validated JAG1-NOTCH1 interface-selective drug. Such candidates require binding-site characterization, target-engagement assays and testing of tumor selectivity. Clinical efficacy and long-term neurological safety remain unestablished.

Safety represents a central translational constraint. Synapsins, Notch signaling and neuregulin pathways have physiological roles in neural development, synaptic function and adult plasticity (9, 20). A therapy that globally perturbs these systems could have cognitive, developmental or seizure-related consequences. A clinically useful strategy would therefore need biomarker selection, local delivery or tumor-state specificity.

Combination strategies may ultimately be required. GBM recurrence reflects GSC persistence, circuit input, immune suppression, invasion and therapy-induced plasticity (2, 33, 34). A differentiation-restoring intervention could weaken the stem-like reservoir without suppressing excitatory input, tumor microtubes or immune evasion. Combinations with radiotherapy, chemotherapy or microenvironmental therapies warrant preclinical evaluation.

## Immune and microenvironmental cross-talk

Glioma integrates neural, immune and stromal signals within a proposed neuro-immune-tumor axis (40). Circuit remodeling can induce regional immunosuppression (41), while hypoxia-associated neuronal activity has been linked to microglial polarization (42). Neuron-glioma communication may therefore reshape the immune microenvironment as well as tumor-cell behavior.

This immune dimension is relevant to Synapsin III for two reasons. First, Notch signaling also shapes immune and stromal cell behavior (20). Second, changing GSC state could alter antigen presentation, cytokine secretion or microglial engagement. These potential immune effects of Synapsin III remain untested.

The most informative next experiments would examine Synapsin III in models that retain relevant microenvironmental components. Patient-derived orthotopic xenografts can test tumor growth and survival but cannot fully resolve immune effects. Syngeneic or humanized models and organoids with immune components could test whether forced *SYN3* expression changes macrophage or microglial states; spatial transcriptomic measurements could complement functional assays.

## Evidence gaps and future directions

The first unresolved question concerns structure-function specificity. The J domain is a candidate explanation for Synapsin III specificity, but its causal contribution remains untested (Figure 1A). Domain-deletion and rescue experiments should establish whether it is required for NRG3 induction, JAG1-NOTCH1 interference, the neuronal-like phenotype and tumor suppression. If required, its interactors and phosphorylation-dependent regulation should be mapped.

The second question is the generalizability of family-wide comparisons. Deng and colleagues already compared *SYN1, SYN2*, and *SYN3* overexpression in matched GSC cultures (19). The next step is independent replication across additional patient-derived states, with isoform-matched expression, endogenous depletion and rescue. Readouts should include protein abundance, differentiation markers, sphere formation, electrophysiology, NRG3 release, Notch activity and *in vivo* tumor initiation. This would distinguish a reproducible family-specific effect from one restricted to the tested overexpression conditions.

The third question is patient stratification. GBM cases differ in circuit dependencies and differentiation programs. Molecular state, anatomical location and neuronal connectivity may influence response (30, 32, 37). Baseline *SYN3* mRNA and Synapsin III protein abundance should be evaluated separately as candidate biomarkers; neither is currently a validated predictor of benefit from a Synapsin III-directed intervention.

The fourth question is therapeutic delivery and durability. AAV9-mediated *SYN3* delivery has provided a preclinical proof of concept (19), but infiltrative GBM requires coverage of invasive margins as well as the tumor core (33, 34). Local sustained-release approaches also warrant investigation. Longitudinal studies must test persistence of the phenotype, recurrent tumor initiation, off-target neural effects and response after treatment withdrawal.

Field-level reviews emphasize malignant circuit integration while recognizing variation across tumor types and anatomical contexts (43–45). Individual proteins illustrate this diversity: Synaptosomal-associated protein 25 (SNAP25) has a reported suppressive role linked to glutaminolysis (46), whereas oncogenic regulation of NLGN3 can support GBM progression (47). Mechanism and tumor state remain more informative than neuronal annotation alone.

## Conclusion

Functional divergence within the synapsin family is an emerging model in GBM. Initial comparisons in patient-derived GSCs distinguish Synapsin III from Synapsin I and II, linking a neuronal-like differentiated phenotype to reduced proliferation and sphere formation (19). The reported mechanism involves NRG3-mediated interference with JAG1-NOTCH1 signaling (Figure 1C). Together, these findings position Synapsin III as a candidate regulator of GSC state, with the extent and durability of its effects across GBM states requiring independent validation (Figure 2).

**Figure 2:**
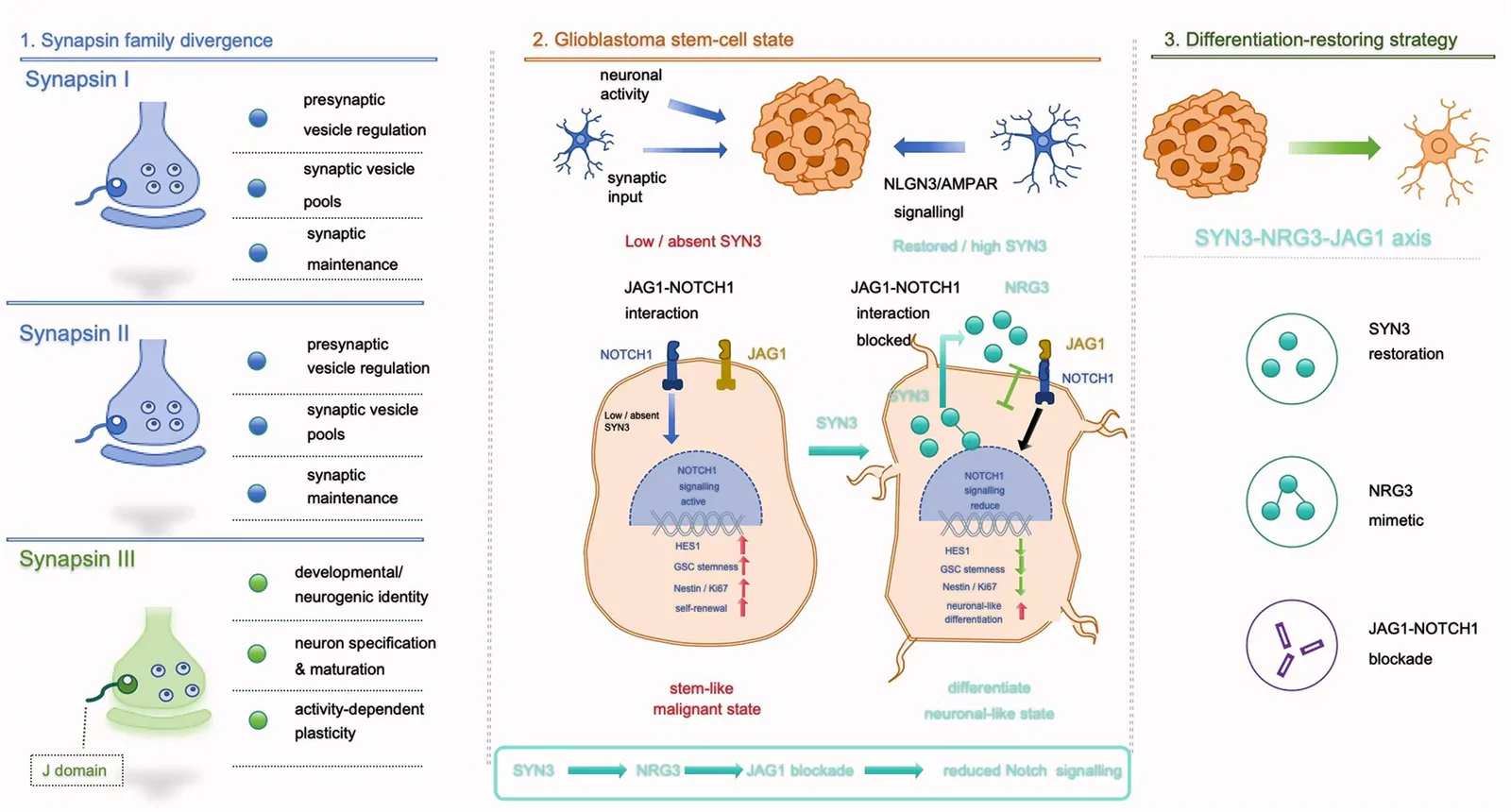
Functional divergence of the synapsin family in glioma: Synapsin Ill as a candidate differentiation-restoring node.

Neuronal proteins can participate in growth-promoting circuit integration or in differentiation-associated suppression of stemness (3, 4, 19). Synapsin III makes this distinction experimentally tractable. Its translational value will depend on domain-specific causality, durable loss of tumor-initiating capacity, predictive patient-state markers and delivery strategies that preserve physiological neural function.
